# Supplementary material for: Enhancement of enzymatic activity by biomolecular condensates through pH buffering
Source: Nat Commun. 2025 Jul 10;16:6368. doi: 10.1038/s41467-025-61013-8 (PMC12246476; doi:10.1038/s41467-025-61013-8)
Supplement: Supplementary file 1 — Supplementary Information [file 41467_2025_61013_MOESM1_ESM.pdf]

# Supplementary Information:

# Enhancement of Enzymatic Activity by Biomolecular Condensates through pH Buffering

F. Stoffel[1], M. Papp[1], M. Gil-Garcia[1], A. M. Küffner[1], A. I. Benítez-Mateos[1], R. P. B. Jacquat[1], N. Galvanetto[2], L. Faltova[1], P. Arosio[1,*]

[1] Department of Chemistry and Applied Biosciences, Institute of Chemical and Bioengineering, ETH Zurich, Zurich, 8093, Switzerland

[2] Department of Biochemistry, University of Zurich, Zurich, 8057, Switzerland

*Corresponding author: paolo.arosio@chem.ethz.ch

| Protein | Sequence |
|---|---|
| BTL2 | MGSSHHHHHHSSGLVPRGSHMASPRANDAPIVLLHGFTGWGREEMLGFKYWGGVRGDIEQWL NDNGYRTYTLAVGPLSSNWDRACEAYAQLVGGTVDYGAAHAAKHGHARFGRTYPGLLPELKRGG RVHIIAHSQGGQTARMLVSLLENGSQEEREYAKAHNVSLSPLFEGGHHFVLSVTTIATPHDGTTLVN MVDFTDRFFDLQKAVLKAAAVASNVPYTSQVYDFKLDQWGLRRQPGESFDHYFERLKRSPVWTST DTARYDLSIPGAEKLNQWVQASPNTYYLSFSTERTHRGALTGNYYPELGMNAFSAVVCAPFLGSYR NEALGIDDRWLENDGIVNTVSMNGPKRGSSDRIVPYDGTLKKGVWNDMGTCNVDHLEVIGVDP NPSFDIRAFYLRLAEQLASLRP |
| Laf1-BTL2-Laf1 | MGSSHHHHHHSSGLVPRGSHMESNQSNNGGSGNAALNRGGRYVPPHLRGGDGGAAAAASAG GDDRRGGAGGGGYRRGGGNSGGGGGGGYDRGYNDNRDDRDNRGGSGGYGRDRNYEDRGYN GGGGGGGNRGYNNNRGGGGGGYNRQDRGDGGSSNFSRGGYNNRDEGSDNRGSGRSYNNDR RDNGGDGASPRANDAPIVLLHGFTGWGREEMLGFKYWGGVRGDIEQWLNDNGYRTYTLAVGPL SSNWDRACEAYAQLVGGTVDYGAAHAAKHGHARFGRTYPGLLPELKRGGRVHIIAHSQGGQTAR MLVSLLENGSQEEREYAKAHNVSLSPLFEGGHHFVLSVTTIATPHDGTTLVNMVDFTDRFFDLQKA VLKAAAVASNVPYTSQVYDFKLDQWGLRRQPGESFDHYFERLKRSPVWTSTDTARYDLSIPGAEKL NQWVQASPNTYYLSFSTERTHRGALTGNYYPELGMNAFSAVVCAPFLGSYRNEALGIDDRWLEND GIVNTVSMNGPKRGSSDRIVPYDGTLKKGVWNDMGTCNVDHLEVIGVDPNPSFDIRAFYLRLAEQ LASLRPMESNQSNNGGSGNAALNRGGRYVPPHLRGGDGGAAAAASAGGDDRRGGAGGGGYRR GGGNSGGGGGGGYDRGYNDNRDDRDNRGGSGGYGRDRNYEDRGYNGGGGGGGNRGYNNN RGGGGGGYNRQDRGDGGSSNFSRGGYNNRDEGSDNRGSGRSYNNDRRDNGGDG |
| Laf1 | MGSSHHHHHHSSGLVPRGSHMESNQSNNGGSGNAALNRGGRYVPPHLRGGDGGAAAAASAG GDDRRGGAGGGGYRRGGGNSGGGGGGGYDRGYNDNRDDRDNRGGSGGYGRDRNYEDRGYN GGGGGGGNRGYNNNRGGGGGGYNRQDRGDGGSSNFSRGGYNNRDEGSDNRGSGRSYNNDR RDNGGDG |

| | |
|---|---|
| DDX4-BTL2-DDX4 | MGSSHHHHHHSSGLVPRGSHMGDEDWEAEINPHMSSYVPIFEKDRYSGENGDNFNRTPASSSE<br>MDDGPSRRDHFMKSGFASGRNFGNRDAGECNKRDNTSTMGGFGVGKSFGNRGFSNSRFEDGD<br>SSGFWRESSNDCEDNPTRNRGFSKRGGYRDGNNSEASGPYRRGGRGSFRGCRGGFGLGSPNNDL<br>DPDECMQRTGGLFGSRRPVLSGTGNGDTSQSRSGSGSERGGYKGLNEEVITGSGKNSWKSEAEG<br>GESASPRANDAPIVLLHGFTGWGREEMLGFKYWGGVRGDIEQWLNDNGYRTYTLAVGPLSSNW<br>DRACEAYAQLVGGTVDYGAAHAAKHGHARFGRTYPGLLPELKRGGRVHIIAHSQGGQTARMLVS<br>LLENGSQEEREYAKAHNVSLSPLFEGGHHFVLSVTTIATPHDGTTLVNMVDFTDRFFDLQKAVLKA<br>AAVASNVPYTSQVYDFKLDQWGLRRQPGESFDHYFERLKRSPVWTSTDTARYDLSIPGAEKLNQW<br>VQASPNTYYLSFSTERTHRGALTGNYYPELGMNAFSAVVCAPFLGSYRNEALGIDDRWLENDGIVN<br>TVSMNGPKRGSSDRIVPYDGTLKKGVWNDMGTCNVDHLEVIGVDPNPSFDIRAFYLRLAEQLASL<br>RPGDEDWEAEINPHMSSYVPIFEKDRYSGENGDNFNRTPASSSEMDDGPSRRDHFMKSGFASGR<br>NFGNRDAGECNKRDNTSTMGGFGVGKSFGNRGFSNSRFEDGDSSGFWRESSNDCEDNPTRNRG<br>FSKRGGYRDGNNSEASGPYRRGGRGSFRGCRGGFGLGSPNNDLDPDECMQRTGGLFGSRRPVLS<br>GTGNGDTSQSRSGSGSERGGYKGLNEEVITGSGKNSWKSEAEGGES |
| AAOx | MGSSHHHHHHSSGLVPRGSHMATNLPTADFDYVVVGAGNAGNVVAARLTEDPDVSVLVLEAGV<br>SDENVLGAEAPLLAPGLVPNSIFDWNYTTTAQAGYNGRSIAYPRGRMLGGSSSVHYMVMMRGST<br>EDFDRYAAVTGDEGWNWDNIQQFVRKNEMVVPPADNHNTSGEFIPAVHGTNGSVSISLPGFPTP<br>LDDRVLATTQEQSEEFFFNPDMGTGHPLGISWSIASVGNGQRSSSSTAYLRPAQSRPNLSVLINAQ<br>VTKLVNSGTTNGLPAFRCVEYAEQEGAPTTTVCAKKEVVLSAGSVGTPILLQLSGIGDENDLSSVGID<br>TIVNNPSVGRNLSDHLLLPAAFFVNSNQTFDNIFRDSSEFNVDLDQWTNTRTGPLTALIANHLAWL<br>RLPSNSSIFQTFPDPAAGPNSAHWETIFSNQWFHPAIPRPDTGSFMSVTNALISPVARGDIKLATSN<br>PFDKPLINPQYLSTEFDIFTMIQAVKSNLRFLSGQAWADFVIRPFDPRLRDPTDDAAIESYIRDNANTI<br>FHPVGTASMSPRGASWGVVDPDLKVKGVDGLRIVDGSILPFAPNAHTQGPIYLVGKQGADLIKAD<br>Q |

**Supplementary Figure 1: Amino acid sequences of BTL2, Laf1-BTL2-Laf1, Laf1, AAOx, and DDX4-BTL2-DDX4 proteins.** The N-terminal His-Tag is highlighted in red, and the IDRs in the chimeric proteins in grey.

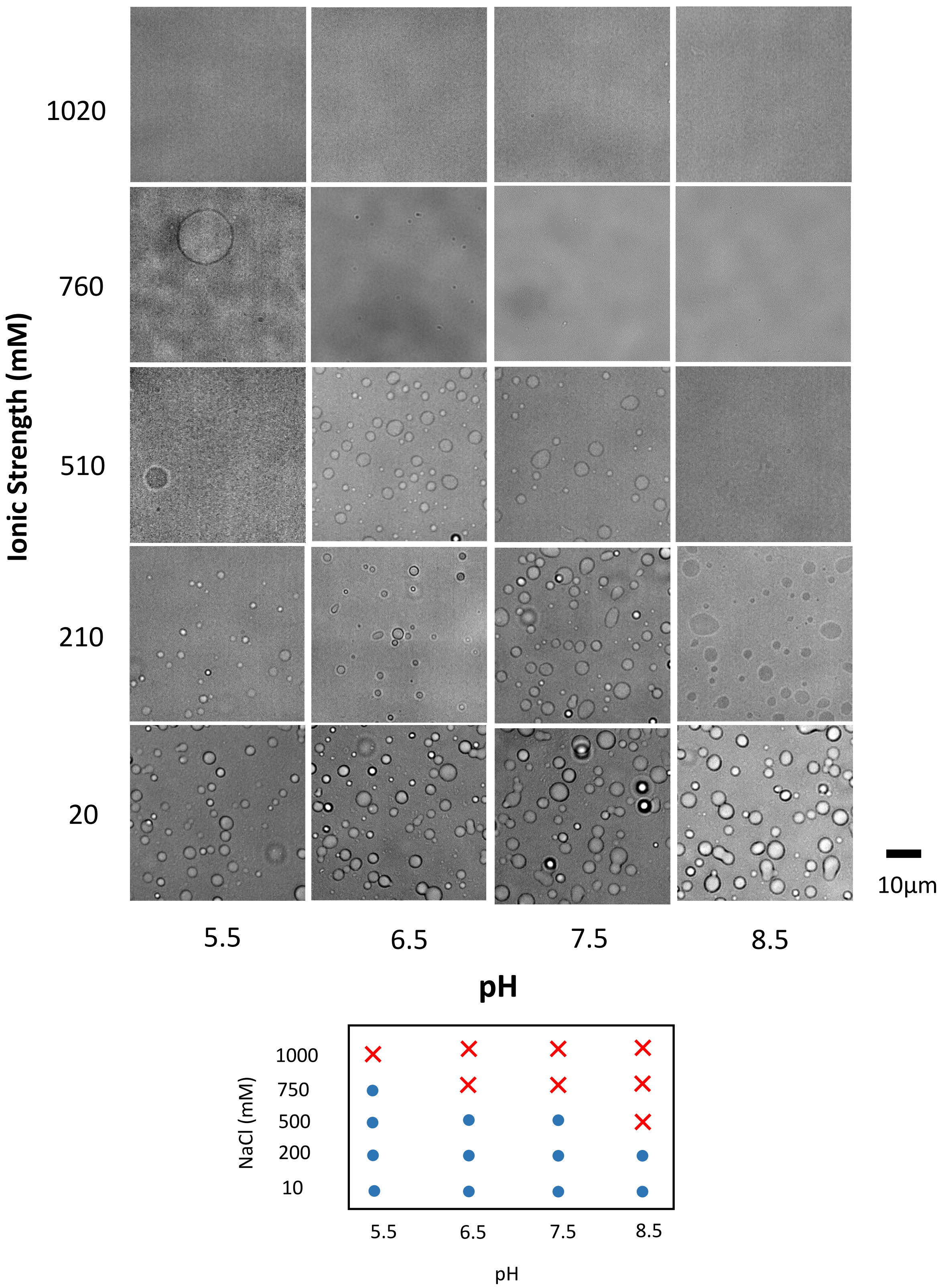


**Supplementary Figure 2: Phase diagram of Laf1-BTL2-Laf1 condensates.** Microscopy analysis of 0.5 µM Laf1-BTL2-Laf1 in 10 mM ionic strength Tris / Bis-tris buffers. Images were taken 10 mins after sample preparation. Contrast and zoom factors were optimized for visualization purposes.

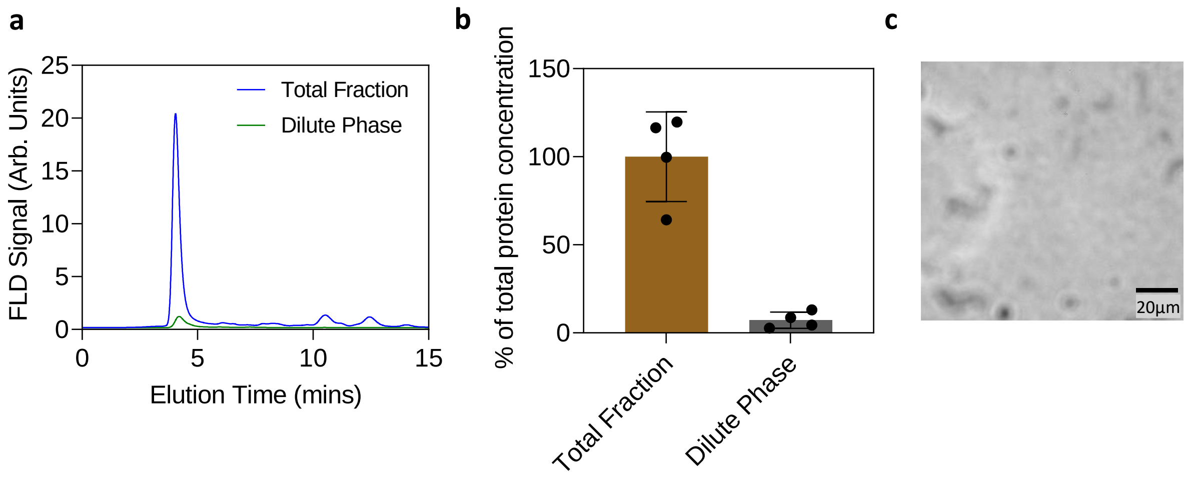


**Supplementary Figure 3: Analysis of enzyme partitioning in the dense phase via size exclusion chromatography**: **A)** SEC chromatograms recorded with the intrinsic fluorescence signal of the protein for a homogeneous Laf1-BTL2-Laf1 solution at 0.5 µM and for the dilute phase in the phase separated system (see Materials and Methods). Curves represent the average of four replicates. **B)** Normalized amount of protein in the dilute phase determined by integration of main peaks corresponding to Laf1-BTL2-Laf1 in the chromatograms shown in panel **A. C)** Bright-field image of the dilute phase separated from the dense phase by centrifugation.

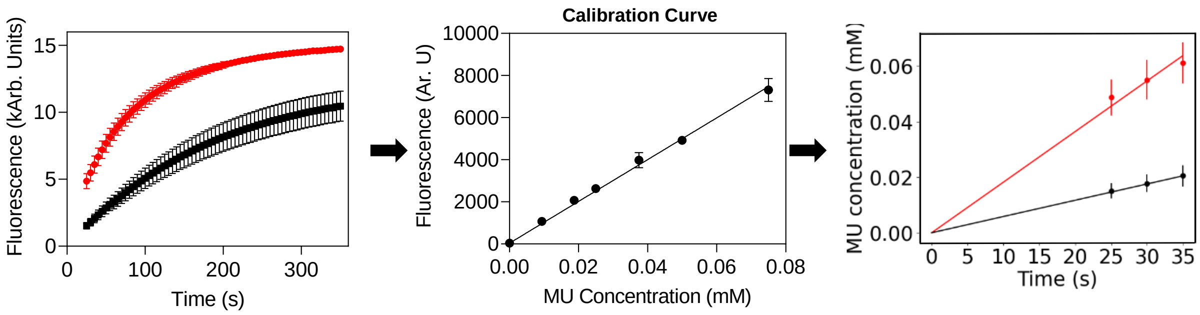


**Supplementary Figure 4: Process to determine initial rates** from the complete kinetic profiles of the hydrolysis catalysed by BTL2 (black) and Laf1-BTL2-Laf1 (red). Error bars denote the standard error of the mean. Linear Regressions were fitted with Graphpad Prism.

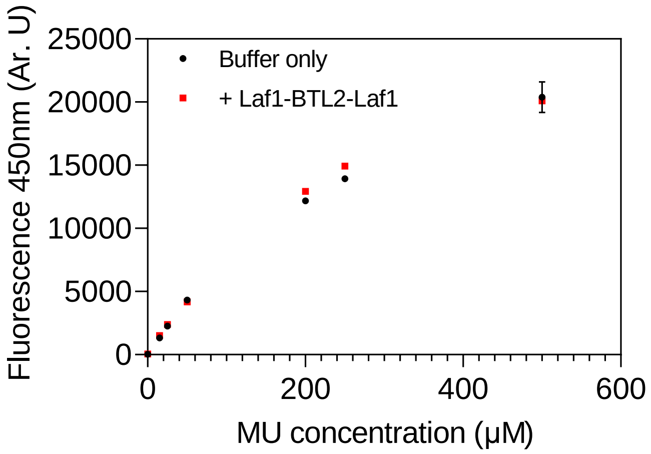


**Supplementary Figure 5: Effect of Laf1-BTL2-Laf1 condensates on MU Fluorescence** MU Fluorescence at 450 nm in 24 mM Tris buffer, 10 mM NaCl, pH 7.5 in the presence (red) and absence (black) of Laf1-BTL2-Laf1 condensates (total protein concentration: 0.5 µM) Samples were measured in triplicate (the Buffer only sample with 500µM MU was measured only in duplicate). Error bars indicate the standard error of the mean.

**a** **b**

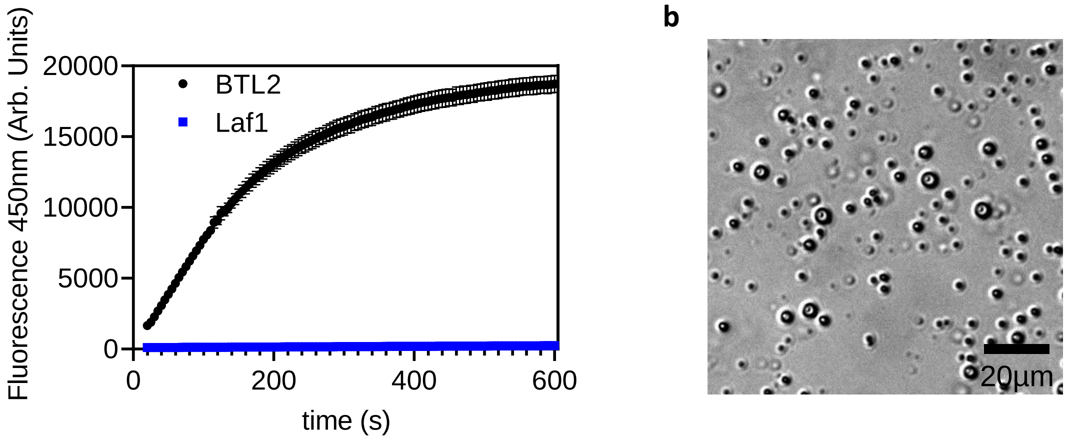


**Supplementary Figure 6: Effect of Laf1 condensates on MUB hydrolysis A)** MUB hydrolysis over time in presence of BTL2 (black symbols, 0.5 µM protein, 0.1 mM MUB) and Laf1 IDR condensates (blue symbols, 10 µM protein, 0.1 mM MUB) in 24 mM Tris buffer at pH 7.5 and 30 mM NaCl, 30 mM Urea. BTL2 and Laf1 condensates were each measured in independent triplicates. **B)** Brightfield microscopy image of Laf1 IDR condensates before adding the substrate for the experiment shown in panel **A.**

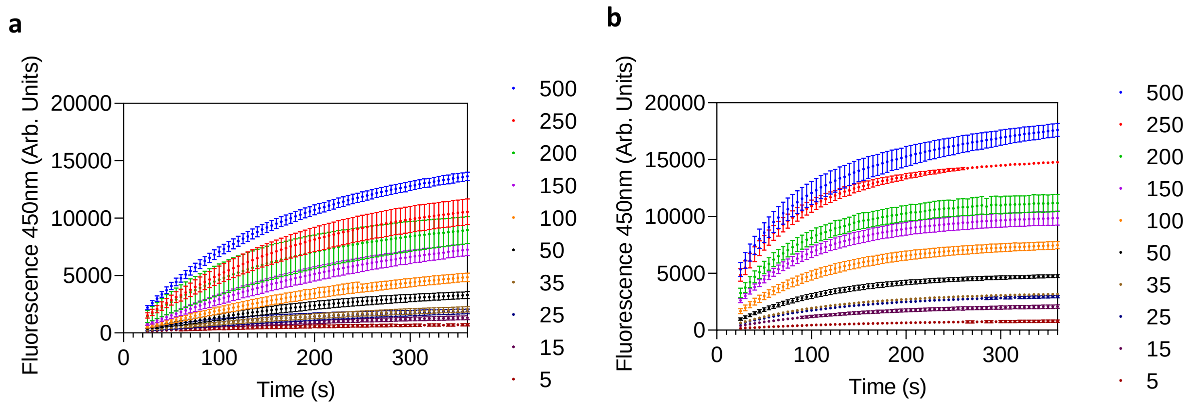


**Supplementary Figure 7: Kinetic curves of MUB hydrolysis for Michaelis Menten Analysis.** Kinetic curve of MUB hydrolysis catalysed by homogenous BTL2 **(A)** and Laf1-BTL2-Laf1 condensates **(B)** at different MUB substrate concentrations (indicated in µM concentrations) in 24 mM Tris, 10 mM NaCl, pH 7.5. For each condition, four (150µM, 100 µM, 50 µM, 35 µM, 25 µM, 15 µM, 5 µM) or five (500 µM, 250 µM, 200 µM for both, 150 µM, 100 µM, and 50 µM for BTL2) independently prepared samples were analysed. Error bars indicate standard error of the mean.

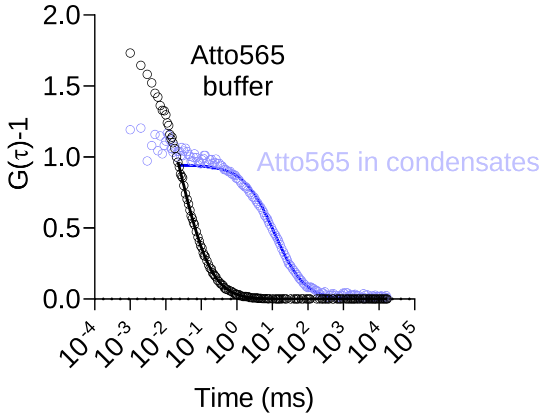


**Supplementary Figure 8: FCS Analysis of Laf1-BTL2-Laf1 condensates** Fluorescence autocorrelation curves for Atto565 in buffer (24 mM Tris, pH 7.5) and within Laf1-BTL2-Laf1 condensates (total protein concentration 0.5 µM).

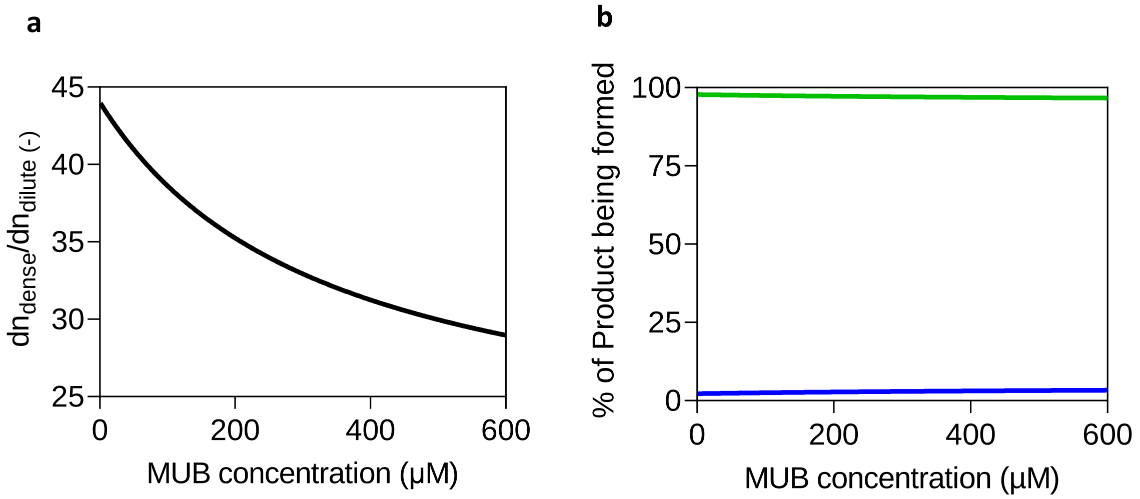


**Supplementary Figure 9: *In silico* Analysis of Product formation. a)** Ratio of product formation in the dense and dilute phases as predicted following Equation 2. **b)** Fraction of product formed in the dense and dilute phases, evaluated from equation (2)

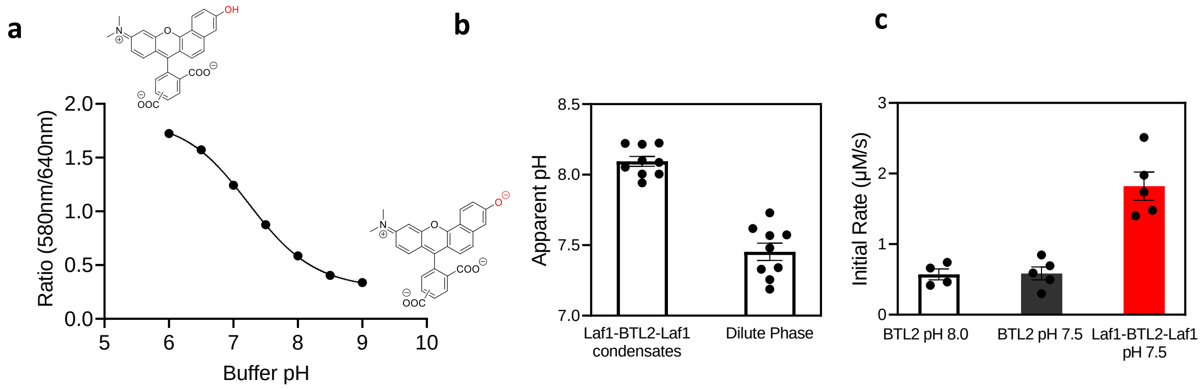


**Supplementary Figure 10: Analysis of pH effects within Laf1-BTL2-Laf1 condensates a)** SNARF-1 calibration curve with associated protonation states. All Tris/ BisTris buffers have 10 mM ionic strength. Each buffer condition was measured in triplicate. **b)** Apparent pH within Laf1-BTL2-Laf1 condensates compared to the dilute BTL2 phase measured with the SNARF1 assay with three independent replicates each measured three times (0.5 µM protein in 24 mM Tris Buffer at pH 7.5 and 10 mM NaCl). **c)** Initial rate of MUB hydrolysis catalyzed by BTL2 at pH 7.5 (5 replicates) and pH 8.0 (4 replicates) compared to that of Laf1-BTL2-Laf1 at pH 7.5 (5 replicates) (0.5 µM protein in 10mM ionic strength and 10mM NaCl). For all conditions independent samples were measured.

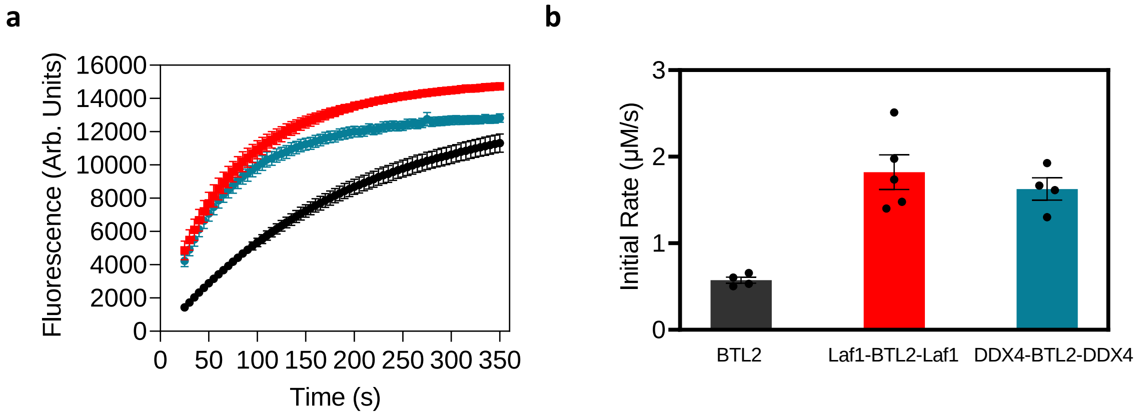


**Supplementary Figure 11: Comparison of lipase activity in BTL2, Laf1-BTL2-Laf1 and DDX4-BTL2-DDX4 systems a)** Kinetic curves of MUB hydrolysis catalyzed by BTL2 (black, 4 replicates), heterogenous Laf1-BTL2-Laf1 (red, 5 replicates), and heterogenous DDX4-BTL2-DDX4 (teal, 4 replicates) (0.5 µM protein and 0.25mM MUB in 24 mM Tris buffer at pH 7.5, with 10 mM NaCl for Laf1-BTL2-Laf1 and BTL2, and 30 mM NaCl for DDX4-BTL2-DDX4) Error bars represent the standard error of the mean of the independently measured samples. **b)** Initial rates extracted from the kinetic curves shown in panel **a.**

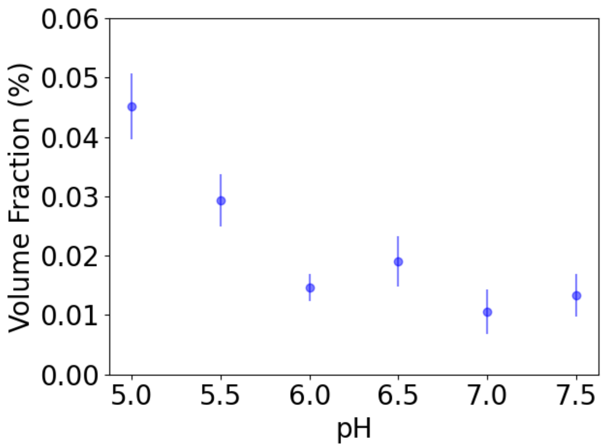


**Supplementary Figure 12: Volume fraction of the dense phase of DDX4-BTL2-DDX4 condensates at different pH values** Volume fractions were determined by z-stack analysis using confocal microscopy for a 0.5µM protein sample in 10mM ionic strength Tris or BisTris buffers and 30 mM NaCl. Eight (pH 5.0, pH 5.5) or six (pH 6.0, pH 6.5, pH 7.0, pH 7.5) independent samples were measured per condition. Error bars denote the standard error of the mean.

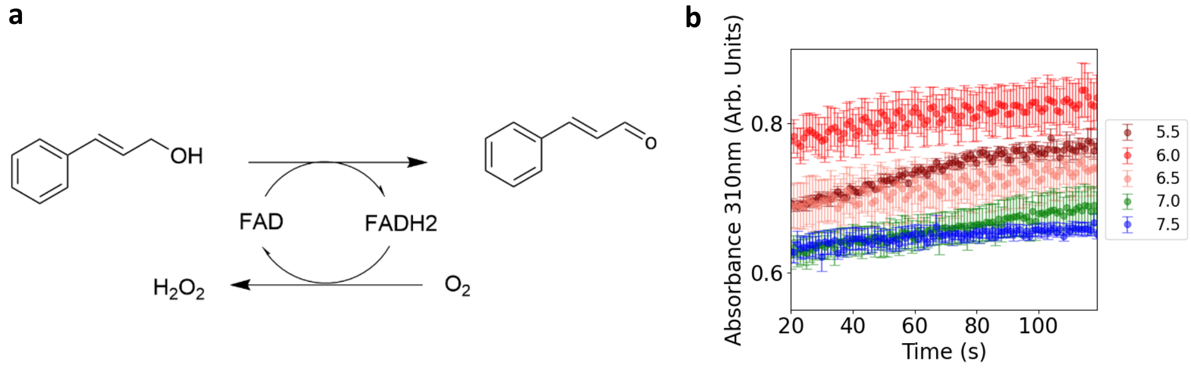


**Supplementary Figure 13: Oxidation reaction catalysed by AAOx. a)** Scheme of the oxidation reaction of CAlc to CAld by AAOx . **b)** Representative kinetic profiles of CAlc oxidation catalyzed by AAOx measured by monitoring the absorbance of the product (310 nm) across different pH values (5 nM enzyme in 10mM ionic strenght Tris/ Bis-Tris buffers). Each condition was measured with three independent samples. Error bars represent the standard error of the mean.

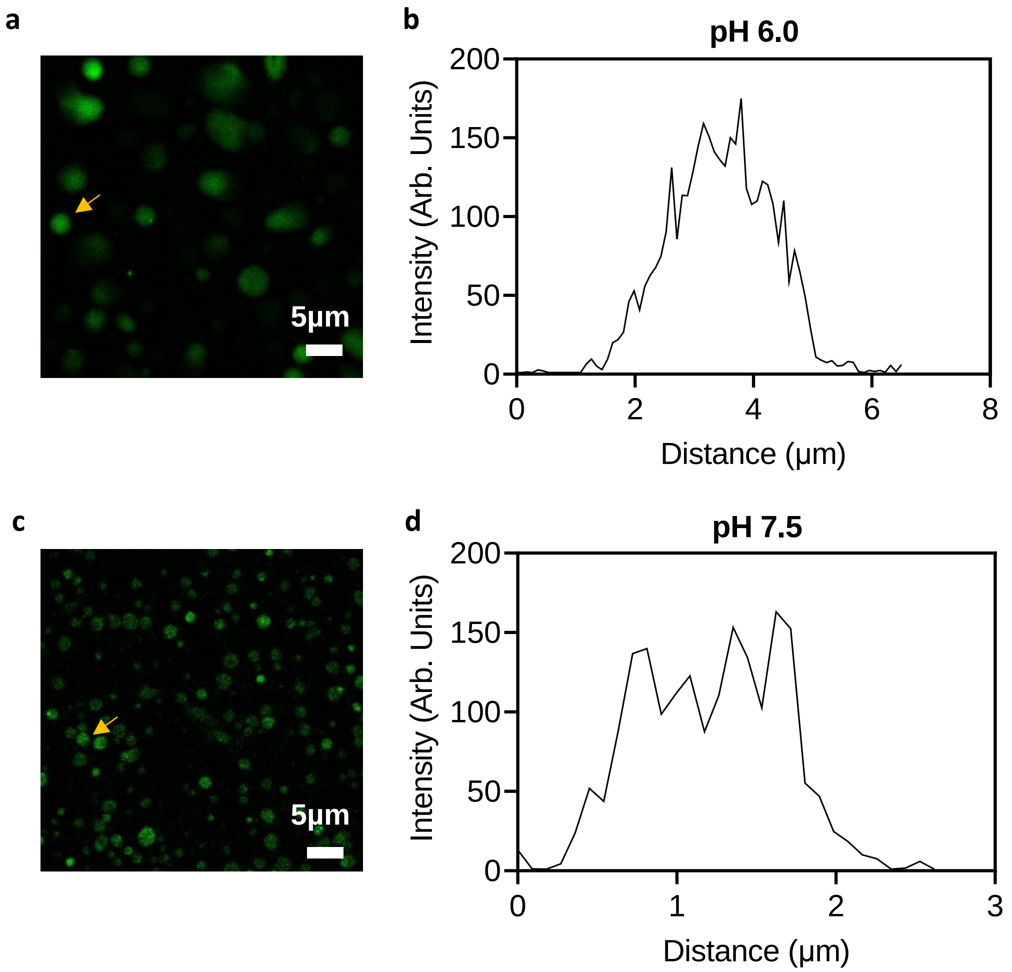


**Supplementary Figure 14: Analysis of AAOx partitioning into DDX4-BTL2-DDX4 condensates** Representative confocal microscopy images and respective fluorescence intensity profiles showing the uptake of AAOx-ATTO565 into DDX4-BTL2-DDX4 condensates at pH 6.0 **(a, b)** and pH 7.5 **(c, d)** in 10mM ionic strength Tris buffers. Data were extracted using ImageJ (see Materials and Methods). Representative analysed condensates are indicated with a yellow arrow.

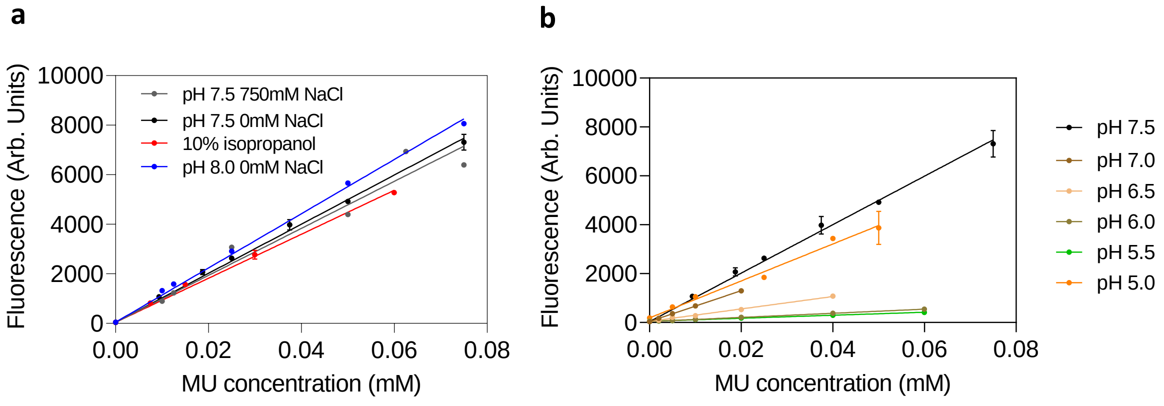


**Supplementary Figure 15: Calibration curves of fluorescence intensity versus MU concentration** Calibration curves used in the measurement of the initial rate of the reaction in different buffer conditions (**a**) Figure 1 in the main text and Supplementary Figure 10; **b**) Figure 3 in the main text and Supplementary Figure 11). For the 10% isopropanol condition, the remaining 90% consists of Tris buffer at pH 7.5. Buffers at different pHs consist of 10 mM ionic strength Tris or BisTris buffers. Error bars denote the standard error of the mean.

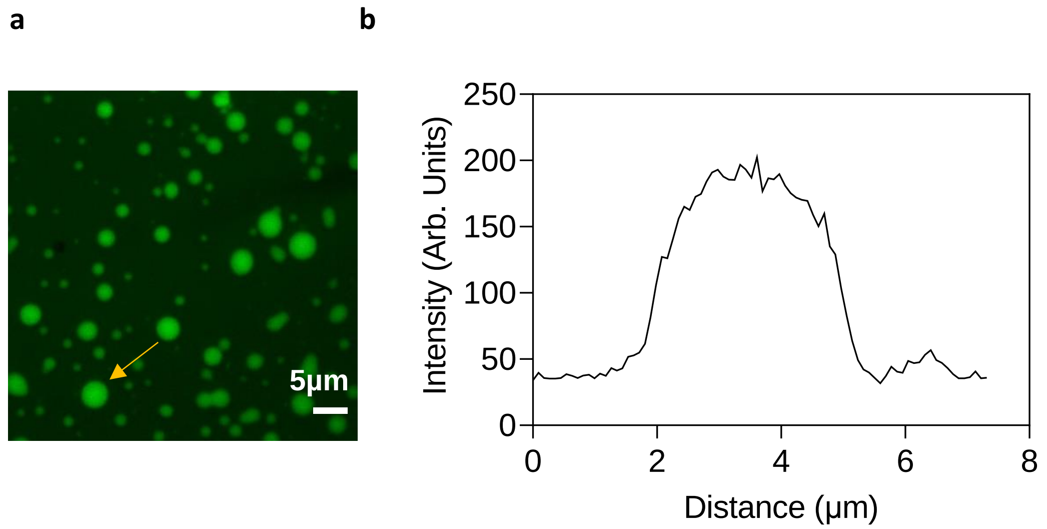


**Supplementary Figure 16: Partitioning of Resorufin into Laf1-BTL2-Laf1 condensates. a:** Representative fluorescence confocal microscopy image of 0.5µM Laf1-BTL2-Laf1 condensates stained with 50 µM Resorufin in 24 mM Tris buffer at pH 7.5 and 10 mM NaCl. The yellow arrow indicates the condensate corresponding to the fluorescence intensity profile shown in panel **b**.

## Kinetic analysis

The following reaction scheme was assumed in both the dilute and dense phases:

$$E + S \rightleftharpoons ES \rightarrow P \tag{3}$$

The two phases were assumed to be at equilibrium. Therefore, phase equilibrium was combined with chemical equilibrium within each phase using activities to describe the rate of product formation.[1]

$$r = k_{cat} \cdot a_{ES} \tag{4}$$

At steady-state conditions, the following equations apply:

$$k_1 \cdot a_E \cdot a_S = (k_{-1} + k_{cat}) \cdot a_{ES} = (k_{-1} + k_{cat}) \cdot \gamma_{ES} \cdot [ES] \tag{5}$$

$$a_E = \gamma_E \cdot ([E]_0 - [ES]) \tag{6}$$

$$[ES] = \frac{[E]_0 \cdot a_S}{K_M \cdot \frac{\gamma_{ES}}{\gamma_E} + a_S} \tag{7}$$

where $K_M = \frac{k_{-1}+k_{cat}}{k_1}$ is the Michaelis–Menten constant. Due to low substrate partitioning, $K_S = 6.3$, as measured with a proxy small molecule, Resorufin (see Method Section and Supplementary Figure 16), the substrate concentration is low in both phases, and therefore $\frac{\gamma_{ES}}{\gamma_E}$ can be considered independent of the substrate concentration. In this case, the rate of product formation in each phase can be expressed as Michaelis–Menten-like kinetics using $a_S$ and $K_M^* = K_M \cdot \frac{\gamma_{ES}}{\gamma_E}$.

$$r = k_{cat} \cdot \gamma_{ES} \cdot [E]_0 \cdot \frac{a_S}{K_M^* + a_S} \tag{8}$$

In the two-phase system, the total rate of product formation is evaluated as:

$$r_{het} = (1 - \Phi_D) \cdot k_{cat}^I \cdot \gamma_{ES}^I \cdot [E]_0^I \cdot \frac{a_S^I}{K_M^{*I} + a_S^I} + \Phi_D \cdot k_{cat}^{II} \cdot \gamma_{ES}^{II} \cdot [E]_0^{II} \cdot \frac{a_S^{II}}{K_M^{*II} + a_S^{II}} \tag{9}$$

where $\Phi_D$ is the volume fraction of the dense phase, and upper indices $I$ and $II$ denote the dilute and the dense phases, respectively. The reaction rate in the homogeneous system is:

$$r_{hom} = k_{cat}^{hom} \cdot \gamma_{ES}^{hom} \cdot [E]_0^{hom} \cdot \frac{a_S^{hom}}{K_M^{*hom} + a_S^{hom}} = k_{cat}^I \cdot \gamma_{ES}^I \cdot [E]_0^{hom} \cdot \frac{a_S^I}{K_M^{*I} + a_S^I} = k_{cat}^I \cdot \gamma_{ES}^I \cdot \frac{[E]_0}{\xi} \cdot \frac{a_S^I}{K_M^{*I} + a_S^I} \tag{10}$$

$k_{cat}$ and $K_M^*$ in the dilute phase are the same for the wild type enzyme and the chimeric protein. Moreover, $\xi = \frac{[E]_0^I}{[E]_0^{hom}} = \frac{1}{K_E \cdot \Phi_D + 1 - \Phi_D}$, where $K_E = \frac{[E]_0^{II}}{[E]_0^I}$ is the enzyme partition coefficient.

We can express the ratio of the reaction rate in the heterogeneous and homogeneous system as:

$$\frac{r_{het}}{r_{hom}} = \frac{(1-\Phi_D)\cdot k_{cat}^{I}\cdot\gamma_{ES}^{I}\cdot[E]_0^{I}\cdot\frac{a_S^{I}}{K_M^{*I}+a_S^{I}}+\Phi_D\cdot k_{cat}^{II}\cdot\gamma_{ES}^{II}\cdot[E]_0^{II}\cdot\frac{a_S^{II}}{K_M^{*II}+a_S^{II}}}{k_{cat}^{I}\cdot\gamma_{ES}^{I}\cdot\frac{[E]_0}{\xi}\cdot\frac{a_S^{I}}{K_M^{*I}+a_S^{I}}} \tag{11}$$

Since $a_S^I = \gamma_S^I \cdot [S]^I$ and likewise $a_S^{II} = \gamma_S^{II} \cdot K_S \cdot [S]^I$,

$$\frac{r_{het}}{r_{hom}} = \frac{(1-\Phi_D)\cdot k_{cat}^{I}\cdot\gamma_{ES}^{I}\cdot[E]_0^{I}\cdot\frac{\gamma_S^I\cdot[S]^I}{K_M^{*I}+\gamma_S^I\cdot[S]^I}+\Phi_D\cdot k_{cat}^{II}\cdot\gamma_{ES}^{II}\cdot[E]_0^{II}\cdot\frac{\gamma_S^{II}\cdot K_S\cdot[S]^I}{K_M^{*II}+\gamma_S^{II}\cdot K_S\cdot[S]^I}}{k_{cat}^{I}\cdot\gamma_{ES}^{I}\cdot\frac{[E]_0}{\xi}\cdot\frac{\gamma_S^I\cdot[S]^I}{K_M^{*I}+\gamma_S^I\cdot[S]^I}} \tag{12}$$

Which can be further simplified as

$$\frac{r_{het}}{r_{hom}} = \xi\cdot(1-\Phi_D)+\xi\cdot\Phi_D\cdot\frac{k_{cat}^{II}}{k_{cat}^{I}}\cdot\frac{\gamma_{ES}^{II}}{\gamma_{ES}^{I}}\cdot K_E\cdot\frac{\frac{K_M^{*I}}{\gamma_S^I}+[S]^I}{\frac{K_M^{*II}}{\gamma_S^{II}\cdot K_S}+[S]^I} \tag{13}$$

Since phase separation is driven by the low complexity domains of the chimeric proteins, we can assume $\gamma_{ES}^I = \gamma_{ES}^{II}$. Moreover, the activity coefficient of the substrate in the dilute phase can be assumed equal to one, leading to equation (1):

$$\frac{r_{het}}{r_{hom}} = \xi\cdot(1-\Phi_D)+\xi\cdot\Phi_D\cdot\frac{k_{cat}^{II}}{k_{cat}^{I}}\cdot K_E\cdot\frac{K_M^{*I}+[S]}{\frac{K_M^{*II}}{\gamma_S^{II}\cdot K_S}+[S]}$$

The values for $K_M^{*I}$, $k_{cat}^I$, $K_E$, $K_S$ and $\Phi_D$ were evaluated experimentally (see section 2.1).

We note that the measured $K_E$ provides the ratio of the chimeric protein concentration in the dense and dilute phases. Since we assume negligible interactions between the intrinsically disordered domain and the substrate, the substrate concentration does not affect the partition coefficient $K_E = \frac{\gamma_{LCD}^I}{\gamma_{LCD}^{II}} = \frac{[E]_0^{II}}{[E]_0^I}\left(= \frac{[E]^{II}+[ES]^{II}}{[E]^I+[ES]^I}\right)$.

$k_{cat}^{II}$ and $\frac{K_M^{*II}}{\gamma_S^{II}\cdot K_S}$ were evaluated from the fitting of $\frac{r_{het}}{r_{hom}}$ at different substrate concentrations according to equation (1), where $r_{het}$ denotes experimental initial reaction rates in the heterogeneous system and $r_{hom}$ corresponds to the fitted Michaelis-Menten rates for the homogeneous system. (Table 1).

We note that in the limit of infinite substrate concentration:

$$\lim_{[S]\to\infty}\left(\frac{r_{het}}{r_{hom}}\right) = \xi\cdot(1-\Phi_D)+\xi\cdot\Phi_D\cdot\frac{k_{cat}^{II}}{k_{cat}^{I}}\cdot K_E \tag{14}$$

From equation (14) $\lim_{[S]\to\infty}\left(\frac{r_{het}}{r_{hom}}\right) > 1$ 1 if and only if $k_{cat}^{II} > k_{cat}^{I}$. This means that if the horizontal asymptote of $\frac{r_{het}}{r_{hom}}$ is larger than one, the maximum rate of the enzymatic reaction is enhanced inside the droplets due to an increase in $k_{cat}$.

The amount of product formed in each phase can be calculated as:

$$\frac{dn_{dense}}{dn_{dilute}} = \frac{\Phi_D \cdot r_{dense}}{(1-\Phi_D)\cdot r_{dilute}} = \frac{\Phi_D \cdot k_{cat}^{II} \cdot \gamma_{ES}^{II} \cdot [E]_0^{II} \cdot \frac{[S]}{\frac{K_M^{*II}}{\gamma_S^{II} \cdot K_S}+[S]}}{(1-\Phi_D)\cdot k_{cat}^{I} \cdot \gamma_{ES}^{I} \cdot [E]_0^{I} \cdot \frac{[S]}{K_M^{*I}+[S]}} = \frac{\Phi_D}{1-\Phi_D} \cdot \frac{k_{cat}^{II}}{k_{cat}^{I}} \cdot K_E \cdot \frac{K_M^{*I}+[S]}{\frac{K_M^{*II}}{\gamma_S^{II} \cdot K_S}+[S]} \tag{15}$$

For the limit of extremely high substrate concentrations:

$$\lim_{[S]\to\infty}\left(\frac{dn_{dense}}{dn_{dilute}}\right) = \frac{\Phi_D}{1-\Phi_D} \cdot \frac{k_{cat}^{II}}{k_{cat}^{I}} \cdot K_E \tag{16}$$

1. Bauermann, J., Laha, S., McCall, P. M., Jülicher, F. & Weber, C. A. Chemical Kinetics and Mass Action in Coexisting Phases. *J. Am. Chem. Soc.* **144**, 19294–19304 (2022).
