# Supplementary material for: Enhancement of enzymatic activity by biomolecular condensates through pH buffering
Source: Nat Commun. 2025 Jul 10;16:6368. doi: 10.1038/s41467-025-61013-8 (PMC12246476; doi:10.1038/s41467-025-61013-8)
Supplement: Supplementary file 2 — Reporting summary [file 41467_2025_61013_MOESM2_ESM.pdf]

nature portfolio

Corresponding author(s): *Paolo Arosio*

Last updated by author(s): *01.05.2025*

Reporting Summary

Nature Portfolio wishes to improve the reproducibility of the work that we publish. This form provides structure for consistency and transparency in reporting. For further information on Nature Portfolio policies, see our Editorial Policies and the Editorial Policy Checklist.

Statistics

For all statistical analyses, confirm that the following items are present in the figure legend, table legend, main text, or Methods section.

n/a	Confirmed	
☐	☒	The exact sample size (*n*) for each experimental group/condition, given as a discrete number and unit of measurement
☐	☒	A statement on whether measurements were taken from distinct samples or whether the same sample was measured repeatedly
☒	☐	The statistical test(s) used AND whether they are one- or two-sided<br>*Only common tests should be described solely by name; describe more complex techniques in the Methods section.*
☒	☐	A description of all covariates tested
☒	☐	A description of any assumptions or corrections, such as tests of normality and adjustment for multiple comparisons
☐	☒	A full description of the statistical parameters including central tendency (e.g. means) or other basic estimates (e.g. regression coefficient) AND variation (e.g. standard deviation) or associated estimates of uncertainty (e.g. confidence intervals)
☒	☐	For null hypothesis testing, the test statistic (e.g. *F*, *t*, *r*) with confidence intervals, effect sizes, degrees of freedom and *P* value noted<br>*Give P values as exact values whenever suitable.*
☒	☐	For Bayesian analysis, information on the choice of priors and Markov chain Monte Carlo settings
☒	☐	For hierarchical and complex designs, identification of the appropriate level for tests and full reporting of outcomes
☒	☐	Estimates of effect sizes (e.g. Cohen's *d*, Pearson's *r*), indicating how they were calculated

Our web collection on statistics for biologists contains articles on many of the points above.

Software and code

Policy information about availability of computer code

Data collection	*ImageJ 2.9.0 was used to aquire all brightfield microscopy images. The Leica Application Suite X (Version 1.0) software was used to aquire confocal images and FCS data. CLARIOstar (5.70 R2) or TECAN SPARKCONTROL (V 3.2) were used for platereader measurements*
Data analysis	*For initial rate determination, GraphPad Prism 9.0.2 was used. To determine kinetic constants in the dense phase a custom mechanistic model code was used using Matlab. For image analysis to determine the hydrophobicity and pH of condensates a custom python code was used. To determine condensate volume fractions a custom python code was used. To analyze Resorufin or AAOx partitioning into condensates ImagJ 2.9.0 was used.*

For manuscripts utilizing custom algorithms or software that are central to the research but not yet described in published literature, software must be made available to editors and reviewers. We strongly encourage code deposition in a community repository (e.g. GitHub). See the Nature Portfolio guidelines for submitting code & software for further information.

Data

Policy information about availability of data

All manuscripts must include a data availability statement. This statement should provide the following information, where applicable:

- Accession codes, unique identifiers, or web links for publicly available datasets
- A description of any restrictions on data availability
- For clinical datasets or third party data, please ensure that the statement adheres to our policy

Unless otherwise stated, all data supporting the results of this study can be found in the article, supplementary, and source data files. The processed data for all plots

in this study are available in the Source Data file. Raw microscopy data for volume fraction analyses, PRODAN microscopy images or Resorufin analyses are available from the corresponding author upon request. The PDB used for the open conformation of BTL2 is 2w22 (https://doi.org/10.2210/pdb2W22/pdb), the PDB used for the closed conformation is 1ji3 (analogue from G. Stearothermophilus) (https://doi.org/10.2210/pdb1JI3/pdb). Source Data are provided with this paper.

Research involving human participants, their data, or biological material

Policy information about studies with human participants or human data. See also policy information about sex, gender (identity/presentation), and sexual orientation and race, ethnicity and racism.

Reporting on sex and gender	*n/a*
Reporting on race, ethnicity, or other socially relevant groupings	*n/a*
Population characteristics	*n/a*
Recruitment	*n/a*
Ethics oversight	*n/a*

Note that full information on the approval of the study protocol must also be provided in the manuscript.

Field-specific reporting

Please select the one below that is the best fit for your research. If you are not sure, read the appropriate sections before making your selection.

☒ Life sciences ☐ Behavioural & social sciences ☐ Ecological, evolutionary & environmental sciences

For a reference copy of the document with all sections, see nature.com/documents/nr-reporting-summary-flat.pdf

Life sciences study design

All studies must disclose on these points even when the disclosure is negative.

Sample size	*At least three individually prepared samples were analysed in each experiment (enzyme kinetics or image analysis). To verify that this is sufficient results from each were compared. If they were comparable, the sample size was deemed large enough.*
Data exclusions	*No data was excluded*
Replication	*At least three individually prepared samples were analysed in each experiment (enzyme kinetics or image analysis). Trends were verified with a biological replicate.*
Randomization	*Randomization was not neccessary in this study, as it is an in vitro study.*
Blinding	*There was no blinding in this study, as it is an in vitro study.*

Behavioural & social sciences study design

All studies must disclose on these points even when the disclosure is negative.

Study description	*n/a*
Research sample	*n/a*
Sampling strategy	*n/a*
Data collection	*n/a*
Timing	*n/a*
Data exclusions	*n/a*
Non-participation	*n/a*
Randomization	*n/a*

Ecological, evolutionary & environmental sciences study design

All studies must disclose on these points even when the disclosure is negative.

Study description	*n/a*
Research sample	*n/a*
Sampling strategy	*n/a*
Data collection	*n/a*
Timing and spatial scale	*n/a*
Data exclusions	*n/a*
Reproducibility	*n/a*
Randomization	*n/a*
Blinding	*n/a*

Did the study involve field work? ☐ Yes ☐ No

Field work, collection and transport

Field conditions	*n/a*
Location	*n/a*
Access & import/export	*n/a*
Disturbance	*n/a*

Reporting for specific materials, systems and methods

We require information from authors about some types of materials, experimental systems and methods used in many studies. Here, indicate whether each material, system or method listed is relevant to your study. If you are not sure if a list item applies to your research, read the appropriate section before selecting a response.

Materials & experimental systems

n/a	Involved in the study
☒	☐ Antibodies
☒	☐ Eukaryotic cell lines
☒	☐ Palaeontology and archaeology
☒	☐ Animals and other organisms
☒	☐ Clinical data
☒	☐ Dual use research of concern
☒	☐ Plants

Methods

n/a	Involved in the study
☒	☐ ChIP-seq
☒	☐ Flow cytometry
☒	☐ MRI-based neuroimaging

Antibodies

Antibodies used	*n/a*
Validation	*n/a*

Eukaryotic cell lines

Policy information about cell lines and Sex and Gender in Research

Cell line source(s)	*n/a*

Authentication	*n/a*
Mycoplasma contamination	*n/a*
Commonly misidentified lines (See ICLAC register)	*n/a*

Palaeontology and Archaeology

Specimen provenance	*n/a*
Specimen deposition	*n/a*
Dating methods	*n/a*

☐ Tick this box to confirm that the raw and calibrated dates are available in the paper or in Supplementary Information.

Ethics oversight	*n/a*

Note that full information on the approval of the study protocol must also be provided in the manuscript.

Animals and other research organisms

Policy information about studies involving animals; ARRIVE guidelines recommended for reporting animal research, and Sex and Gender in Research

Laboratory animals	*n/a*
Wild animals	*n/a*
Reporting on sex	*n/a*
Field-collected samples	*n/a*
Ethics oversight	*n/a*

Note that full information on the approval of the study protocol must also be provided in the manuscript.

Clinical data

Policy information about clinical studies
All manuscripts should comply with the ICMJEguidelines for publication of clinical research and a completedCONSORT checklist must be included with all submissions.

Clinical trial registration	*n/a*
Study protocol	*n/a*
Data collection	*n/a*
Outcomes	*n/a*

Dual use research of concern

Policy information about dual use research of concern

Hazards

Could the accidental, deliberate or reckless misuse of agents or technologies generated in the work, or the application of information presented in the manuscript, pose a threat to:

No	Yes	
[x]	[ ]	Public health
[x]	[ ]	National security
[x]	[ ]	Crops and/or livestock
[x]	[ ]	Ecosystems
[x]	[ ]	Any other significant area

Experiments of concern

Does the work involve any of these experiments of concern:

No	Yes	
[x]	[ ]	Demonstrate how to render a vaccine ineffective
[x]	[ ]	Confer resistance to therapeutically useful antibiotics or antiviral agents
[x]	[ ]	Enhance the virulence of a pathogen or render a nonpathogen virulent
[x]	[ ]	Increase transmissibility of a pathogen
[x]	[ ]	Alter the host range of a pathogen
[x]	[ ]	Enable evasion of diagnostic/detection modalities
[x]	[ ]	Enable the weaponization of a biological agent or toxin
[x]	[ ]	Any other potentially harmful combination of experiments and agents

Plants

Seed stocks — *n/a*

Novel plant genotypes — *n/a*

Authentication — *n/a*

ChIP-seq

Data deposition

- [ ] Confirm that both raw and final processed data have been deposited in a public database such as GEO.
- [ ] Confirm that you have deposited or provided access to graph files (e.g. BED files) for the called peaks.

Data access links
May remain private before publication. — *n/a*

Files in database submission — *n/a*

Genome browser session
(e.g. UCSC) — *n/a*

Methodology

Replicates — *n/a*

Sequencing depth — *n/a*

Antibodies — *n/a*

Peak calling parameters — *n/a*

Data quality — *n/a*

Software — *n/a*

Flow Cytometry

Plots

Confirm that:

- [ ] The axis labels state the marker and fluorochrome used (e.g. CD4-FITC).
- [ ] The axis scales are clearly visible. Include numbers along axes only for bottom left plot of group (a 'group' is an analysis of identical markers).
- [ ] All plots are contour plots with outliers or pseudocolor plots.
- [ ] A numerical value for number of cells or percentage (with statistics) is provided.

Methodology

Sample preparation — n/a

Instrument — n/a

Software — n/a

Cell population abundance — n/a

Gating strategy — n/a

- [ ] Tick this box to confirm that a figure exemplifying the gating strategy is provided in the Supplementary Information.

Magnetic resonance imaging

Experimental design

Design type — n/a

Design specifications — n/a

Behavioral performance measures — n/a

Acquisition

Imaging type(s) — n/a

Field strength — n/a

Sequence & imaging parameters — n/a

Area of acquisition — n/a

Diffusion MRI ☐ Used ☐ Not used

Preprocessing

Preprocessing software — n/a

Normalization — n/a

Normalization template — n/a

Noise and artifact removal — n/a

Volume censoring — n/a

Statistical modeling & inference

Model type and settings — n/a

Effect(s) tested — n/a

Specify type of analysis: ☐ Whole brain ☐ ROI-based ☐ Both

April 2023

Statistic type for inference

(See Eklund et al. 2016)

n/a

Correction

n/a

Models & analysis

n/a	Involved in the study
☐	☐ Functional and/or effective connectivity
☐	☐ Graph analysis
☐	☐ Multivariate modeling or predictive analysis

Functional and/or effective connectivity

n/a

Graph analysis

n/a

Multivariate modeling and predictive analysis

n/a
